# Leptin Manipulation Reduces Appetite and Causes a Switch in Mating Preference in the Plains Spadefoot Toad (*Spea bombifrons*)

**DOI:** 10.1371/journal.pone.0125981

**Published:** 2015-04-28

**Authors:** Nicholas W. Garcia, Karin S. Pfennig, Sabrina S. Burmeister

**Affiliations:** 1 Department of Biology, University of North Carolina, Chapel Hill, North Carolina, United States of America; 2 Curriculum in Neurobiology, University of North Carolina, Chapel Hill, North Carolina, United States of America; University of Missouri, UNITED STATES

## Abstract

Condition- or context-dependent mate choice occurs when females modify their mate preferences depending on their internal or external environment. While the ecological and evolutionary factors that favor the evolution of such plasticity are emerging, relatively little is known of the mechanisms underlying such choice. Here we evaluated whether leptin, a protein hormone involved in the regulation of appetite, might affect the expression of condition-dependent mate choice decisions. To do so, we administered leptin to spadefoot toads, *Spea bombifrons*, which exhibit condition-dependent mate choice for males of their own species versus congeneric males of *S*. *multiplicata*. In particular, poor-condition *S*. *bombifrons* are more likely than are good-condition *S*. *bombifrons* to prefer *S*. *multiplicata* males, but only in environments where hybridization between the two species is beneficial. We found that our leptin treatment reduced appetite in *S*. *bombifrons* adults, as was expected from leptin's known effects on appetite. However, although we predicted that leptin would reduce female preferences for heterospecific males, we found the opposite. In particular, our leptin treatment generated a consistent, repeatable preference for heterospecifics in an environment where females generally prefer conspecifics regardless of condition. These results indicate that leptin has the potential to affect female mate choice, but that it might do so in non-intuitive ways.

## Introduction

For sexually reproducing animals, mate choice is a key decision that influences evolutionary fitness [1]. When choosing mates, females often assess not only prospective mates, but also their own physiological condition, social environment, or the environment in which mating (or offspring development) occurs (reviewed in [2, 3]). The fitness consequences and evolutionary implications of such context- or condition-dependent mate choice are beginning to emerge [2, 3]. Yet, the underlying mechanisms that mediate such choice are less well understood.

Leptin is a peptide hormone best known for its role in maintaining metabolic state through its effects on appetite: across vertebrates, leptin administration reduces food intake [4]. In mammals, but likely not in other vertebrates, leptin appears to have the additional role of signaling adiposity (reviewed in [4]). However, leptin’s effects reach beyond metabolic state, and those effects potentially vary across taxa [4]. Indeed, leptin affects cognitive function and memory formation [5, 6], stress responses [7], immune system activity [8], reproductive maturity [9], and even trade-offs among these functions (e.g., between reproductive investment and immune function [10]). Based on these wide-ranging effects, leptin could affect mate choice either directly (i.e., as a signal of metabolic state or satiety) or indirectly (e.g., through effects on other systems).

Here, we evaluated whether exogenous leptin affects condition-dependent mate choice using the Plains spadefoot toad (*Spea bombifrons*). In part of its range, *S*. *bombifrons* co-occurs and hybridizes with a congener, *S*. *multiplicata*. In this region, female *S*. *bombifrons* discriminate *S*. *bombifrons* calls from *S*. *multiplicata* calls [11]. However, females facultatively alter their preferences for conspecifics depending on their body condition and pond depth (which varies with rainfall in a given year) [11]. Such plasticity in female choice appears to have evolved because hybridization with *S*. *multiplicata* (which is faster developing) is beneficial in shallow water: hybrid tadpoles develop rapidly and are therefore more likely to escape an ephemeral pond [11]. This is especially important for poor-condition females, which produce slower developing tadpoles [11]. Thus, whereas females prefer conspecific calls in deep water (where tadpoles have time to develop), in shallow water, females—particularly those in poor condition—are more likely to prefer *S*. *multiplicata* calls [11]. We hypothesized that, if leptin enhances a female’s perception of her energy levels (e.g., via effects on appetite or perceived body condition), exogenous leptin should reduce preferences for heterospecifics in shallow water.

## Methods

Our specific goals were to: 1) verify the effect of exogenous leptin on appetite to confirm that our treatment elicits predictable physiological effects in *S*. *bombifrons*; 2) test the effect of exogenous leptin on mating preferences in deep and shallow pools.

### Animals and housing

In all experiments, we used adult, sexually mature female *S*. *bombifrons* (mean mass ± SD = 16.47 ± 4.06 g) that were wild-caught from populations that co-occur with the Mexican spadefoot toad (*S*. *multiplicata*) near Portal, Arizona USA. The animals were collected with permission from the State of Arizona Game and Fish Department under the auspices of a scientific collection permit issued to KSP. This species is not endangered or protected. We fed toads live nutrient-dusted crickets *ad libitum*, except for the subjects in the appetite experiment (described below). Females were randomly assigned to treatment groups and mass did not vary between leptin and saline groups in either experiment (mean ± SD in appetite study: leptin = 13.81 ± 3.77 g, saline = 14.75 ± 2.85 g, *t*_17_ = 0.62, *p* = 0.55; mean ± SD in phonotaxis study: leptin = 15.78 ± 4.32 g, saline ± SD = 17.22 ± 4.04 g, *t*_48_ = 1.23, *p* = 0.23). The Institutional Animal Care and Use Committee (IACUC) of the University of North Carolina approved all animal procedures.

### Hormone production and injections

We expressed recombinant leptin in chemically competent *E*. *coli* (BL21 Star (DE3)pLysS, Invitrogen, Carlsbad, CA) using a plasmid construct containing the leptin coding sequence from *Xenopus laevis* (pET151/D-TOPO, Invitrogen, Carlsbad, CA; courtesy of the R. Denver Lab, University of Michigan, Ann Arbor, MI) [12], as follows. We transformed the cells using heat shock and cultured them on selective agarose. Next, we grew a single colony in selective LB broth to OD_600_ = 0.5 and induced leptin expression by adding isopropyl β-D-1-thiogalactopyranoside (IPTG) to a concentration of 0.1 mM, culturing the cells at 37°C for an additional 3 h. These conditions optimized the amount of recombinant leptin produced.

We then purified the hormone using a method adapted from Crespi and Denver [12]. Specifically, we produced whole-cell lysate by boiling spun-down cells in SDS-PAGE prep solution for 3 min and then electrophoresed it on polyacrylamide. We excised and electroeluted the induced peptide from the gel, and dialyzed it against 0.9% saline overnight. The plasmid sequence encodes a poly-histidine tag upstream of the leptin sequence, thus we were able to confirm the identity of recombinant leptin by using a Western blot to identify a poly-histidine tagged-peptide of the expected size: the recombinant *Xenopus* leptin (NCBI accession no. AY884210) plus the poly-histidine and V5 tags produces a 21.6 kD protein (anti-poly-histidine antisera courtesy of the J. Sekelsky Lab, University of North Carolina). Both whole cell lysate and the electroeluted product contained a single poly-histidine positive band near 21 kD (S1 Fig). We used a Coomassie stain to confirm that our electroeluted protein sample included only a single protein band at the expected size (S2 Fig). We then determined the stock leptin concentration using the Bradford reagent.

In each experiment, we gave subcutaneous injections (0.1 ml) of leptin dissolved in saline (2 ng per g body mass of toad) or saline once per day for six sequential days. The sixth injection was given 1 h prior to each behavioral trial. Our dose was modest compared to similar (i.e., subcutaneous) treatments used previously in frogs [12]. Specifically, Crespi and Denver [12] found that 2 μg of leptin per tadpole (corresponding to about 1 μg per gram body weight) reduced weight gain. Unfortunately, assays for amphibian leptin do not exist at this time, so we cannot relate our leptin treatment to endogenous leptin levels.

### Appetite assay

We first examined the effect of our injections on prey-catching behavior as a measure of appetite. One week before trials, females were not fed. Following leptin (n = 9) or saline (n = 9) treatment (as above), we presented each female with approximately 50 crickets in a covered arena (0.6 m x 0.3 m x 0.3 m) and we counted the cumulative attacks made by each toad in 3 min intervals over the course of 15 min.

### Phonotaxis tests

We examined the effects of leptin (n = 30) or saline (n = 20) on mating preferences in two-choice phonotaxis trials using previous methods. Specifically, we placed each female in the center of a circular water-filled wading pool (1.8 m diameter). Each female was initially placed on a central platform (above water level) equidistant between two speakers broadcasting either conspecific or heterospecific calls. The stimuli have been used previously and were composed of average call characteristics for each species [11, 13]. One hour after the final leptin injection (see above), we tested each female in back-to-back trials in shallow (6 cm) and deep (30 cm) pools; the pond depth of the initial trial was randomly assigned for each female to control for order effects. We scored a female as preferring a call stimulus if it approached and touched a speaker. This is a reliable method for assessing mate choice because females initiate mating by closely approaching or touching males [14]. We scored females as non-responsive if they did not choose a stimulus within 30 minutes. We also recorded the latency to choose a call.

Because leptin-treated females preferred heterospecific calls in the deep-water environment (see Results), we asked whether this preference was repeatable by testing an additional group of leptin-treated females (n = 21) in deep water in four trials. We gave the first two tests in back-to-back trials one hour following the last leptin injection, as described above. We then gave the females one week with no treatment before beginning the course of injections again, followed by the last two tests in back-to-back trials. We measured repeatability as the total number of trials in which each female selected the heterospecific call.

### Statistical analysis

To determine if leptin affected appetite, we used a repeated measures ANOVA with hormone treatment as a between-subjects factor, time as a within-subjects factor, and their interaction to detect treatment effects on prey attacks. In the initial phonotaxis experiment, we used contingency table analysis with Fisher’s exact tests to determine if leptin-treated females expressed different patterns of preference from saline-treated females. In addition, to test whether leptin affected latency to choose, we used a mixed effects model with hormone treatment, water level, and their interaction as fixed factors and toad as a random factor; because latency data were non-normally distributed, we first log-transformed them. We also used chi-square tests to determine whether female preferences within either treatment group or water level were significantly different from a random 1:1 expectation. We determined if leptin-treated females consistently preferred heterospecific calls in the repeatability tests using a one-sample *t*-test, assuming females with no preference would randomly select the heterospecific call twice (half the trials). We used JMP (9.0, SAS, Cary NC) for all statistical analyses. Data are available in S1–S3 Tables.

## Results

**Fig 1:**
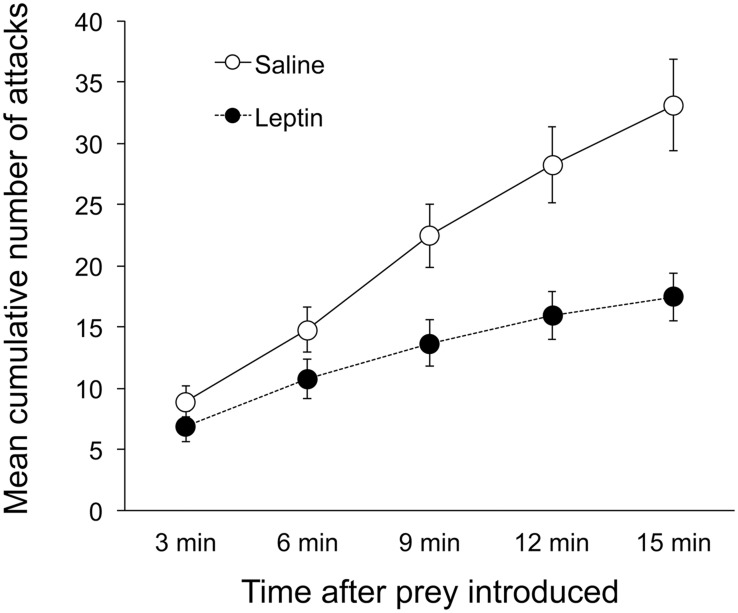
Cumulative attacks (+/- S.E.M.) on cricket prey over time for saline- and leptin-treated female *S*. *bombifrons*. Leptin-treated females attacked prey significantly less than saline-treated females.

We found that leptin-treated toads attacked fewer crickets (*F*_*1*, *16*_ = 8.59, *p* = 0.010) and had a lower rate of attacks (*F*_*4*, *13*_ = 4.23, *p* = 0.021) than saline-treated toads (Fig 1), indicating that leptin reduces appetite in *S*. *bombifrons* as it does in other vertebrates.

**Fig 2:**
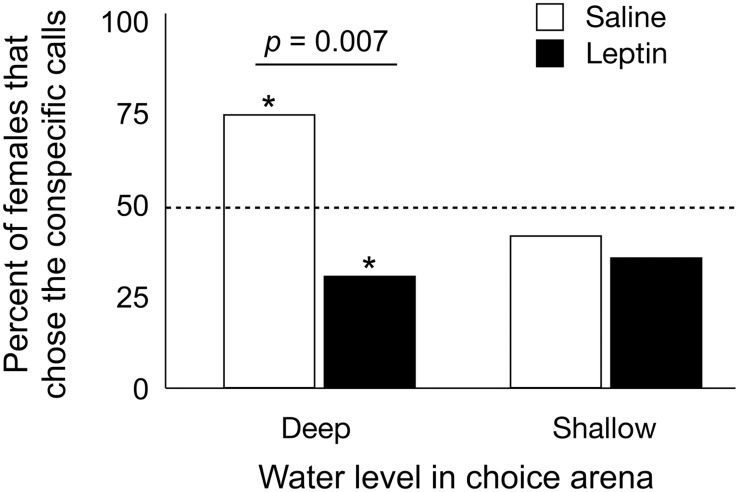
Percentage of female *S*. *bombifrons* that chose conspecific versus heterospecific male calls when treated with saline or leptin. Asterisks indicate significant differences from the random expectation of 50% (indicated by dashed line), while the bar indicates a significant difference in the preferences between saline- and leptin-treated females in deep water.

However, we found that leptin caused unexpected effects on the expression of female mate preferences. In deep water, *S*. *bombifrons* females generally prefer conspecifics [11], and, indeed, our saline-treated toads preferred the conspecific calls under these conditions (log-likelihood chi-square = 5.2, *n* = 20, *p* = 0.022; Fig 2). In contrast, leptin-treated toads preferred heterospecific calls in deep water (log-likelihood chi-square = 3.95, *n* = 26, *p* = 0.047; Fig 2). A direct comparison of preferences in deep water revealed that leptin significantly affected mating preferences (Fisher’s exact test, *p* = 0.007; Fig 2), and there was a trend (*F*_*1*, *44*_ = 3.7, *p* = 0.06) suggesting that leptin-treated females responded more quickly (mean ± SE = 309 ± 44 s) than saline-treated females (mean ± SE = 396 ± 65 s). Importantly, we found that the preferences for heterospecific calls expressed by leptin-treated females in deep water was consistent across four trials (*t*_20_ = 3.16, *p* = 0.005), indicating that the effect of leptin produced a strong, repeatable preference for heterospecific calls. In shallow water, leptin had no detectable effect on mating preferences (Fisher’s exact test, *p* = 0.77).

## Discussion

We predicted that exogenous leptin would induce the expression of mate preferences in female *S*. *bombifrons* that are associated with good condition. However, although leptin reduced appetite in female *S*. *bombifrons* in a manner expected, given leptin’s role in signaling metabolic state, it had an unexpected effect on mating preferences. Specifically, instead of causing females to behave as though they were in good body condition, which would promote conspecific preferences in both shallow and deep pools, we found that leptin caused females to prefer the heterospecific calls in the deep-water environment. Thus, in spadefoot toads, leptin does not appear to mediate condition-dependent preferences by directly linking metabolic state and reproductive decisions.

In addition to its effects on appetite, leptin is known to promote ovarian development. Although we do not know whether ovarian state affects mating preferences in *S*. *bombifrons*, we have no reason to predict that advanced development would promote heterospecific preferences. Indeed, in cases in which advanced ovarian development affects mating preferences, it makes females less choosy (e.g., [15], but it has never before been shown to cause a switch in mating preferences. Although we still have an incomplete understanding of how and why leptin affects mating preferences in *S*. *bombifrons*, our results are notable, as they are the first to show that a hormone can cause a switch in mating preferences.

Could our results represent a pharmacological effect of exogenous leptin? Without additional studies of the relationship between plasma leptin levels and mating preferences, we cannot know how our leptin treatment relates to the natural expression of condition-dependent mate choice in *S*. *bombifrons* (assays for amphibian leptin do not exist at this time). Nonetheless, our results are unlikely to be an artifact of our leptin manipulation for three reasons. First, the recombinant leptin we used has previously been shown to affect appetite and development time in *Xenopus* and *Spea* tadpoles [12] and our dosage was considerably lower, reducing the potential for pharmacological effects. Second, our leptin treatment produced the predicted effect on appetite, indicating that our manipulations had relevant physiological effects. Third, leptin-treated animals behaved normally, eating crickets (albeit fewer) and expressing mate preferences, which they would be unlikely to do if the leptin had simply made them ill. Importantly, even if leptin’s effect on preferences is pharmacological, understanding these mechanisms could provide novel insight into the hormonal and neural control of mate choice plasticity.

What can our results tell us about the function of leptin? For decades, mammalian leptin has been under intense study as potentially playing a role in obesity [15]. However, even within mammals, leptin is a pleiotropic hormone that affects functions as diverse as immune function [8], bone development [16], and synaptic plasticity [5]. Although leptin correlates with fat stores (adiposity) within humans [17] and laboratory mice [18], where levels of adiposity range widely, it is doubtful that leptin is a true signal of adiposity. Indeed, in free ranging mammals [19–21], lizards [22], and fish [23], leptin levels and adiposity do not covary. Our results here suggest that, in the spadefoots as well, leptin does not serve as a simple adipostat on which mate choice decisions depend.

Regardless of the means by which leptin acts, our results suggest the possibility of a novel function for leptin as a potential contributing factor to mate choice decisions. Research on the underlying mechanisms of condition-dependent mate choice is still needed, as is the study of non-mammalian leptins [24]. Identifying the roles that leptin might play during ecologically relevant decisions such as mate choice is potentially crucial to understanding how behavioral plasticity evolves and is expressed across species.

## Supporting Information

S1 Fig Western blot of whole cell lysate (WCL) and electroeluted protein (EP) for recombinant leptin’s poly-histidine tag. Recombinant leptin has a molecular mass of 21.6 kD.
(TIF)

S2 Fig A Coomassie stain of our electroeluted protein (EP) sample shows only a single band of the expected size of 21.6 kD. (TIF)

S1 Table Number of cumulative cricket attacks of each toad following saline or leptin treatment. (TXT)

S2 Table Mating call preference expressed by each toad in the high and low water phonotaxis conditions. (TXT)

S3 Table Number of conspecific call preferences expressed by leptin-treated toads in four sequential tests. (TXT)
