# Supplementary figures and images for: Leptin Manipulation Reduces Appetite and Causes a Switch in Mating Preference in the Plains Spadefoot Toad (Spea bombifrons)
Source: PLoS One. 2015 Apr 28;10(4):e0125981. doi: 10.1371/journal.pone.0125981 (PMC4412710; doi:10.1371/journal.pone.0125981)

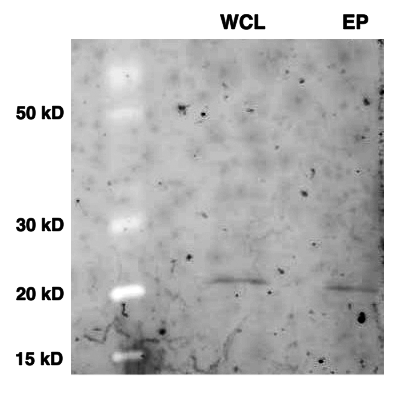

Supplement: S1 Fig — Recombinant leptin has a molecular mass of 21.6 kD. (TIF) [file pone.0125981.s001.tif]
